# Itraconazole, Voriconazole, and Posaconazole CLSI MIC Distributions for Wild-Type and Azole-Resistant *Aspergillus fumigatus* Isolates

**DOI:** 10.3390/jof4030103

**Published:** 2018-08-29

**Authors:** Jochem B. Buil, Ferry Hagen, Anuradha Chowdhary, Paul E. Verweij, Jacques F. Meis

**Affiliations:** 1 Department of Medical Microbiology, Radboud University Medical Center, 6525 Nijmegen, The Netherlands; paul.verweij@radboudumc.nl; 2 Center of Expertise in Mycology Radboudumc/CWZ, 6525 Nijmegen, The Netherlands; jacques.meis@gmail.com; 3 Department of Medical Microbiology and Infectious Diseases, Canisius Wilhelmina Hospital, 6532 Nijmegen, The Netherlands; f.hagen@gmail.com; 4 Department of Medical Mycology, Westerdijk Fungal Biodiversity Institute, 3584 Utrecht, The Netherlands; 5 Department of Medical Mycology, Vallabhbhai Patel Chest Institute, University of Delhi, Delhi 110021, India; chowdhary.anuradha@gmail.com

**Keywords:** *cyp51A*, aspergillosis, azole resistance, ECV, CLSI broth microdilution

## Abstract

Azole resistance in *Aspergillus fumigatus* is most frequently conferred by mutations in the *cyp51A* gene encoding 14α-sterol demethylases. TR_34_/L98H and TR_46_/Y121F/T289A are the two most common mutations associated with environmental resistance selection. We studied the minimal inhibitory concentration (MIC) distribution of clinical *A. fumigatus* isolates to characterize the Clinical and Laboratory Standards Institute (CLSI) susceptibility profiles of isolates with the wild-type (WT) *cyp51A* genotype, and isolates with the TR_34_/L98H and TR_46_/Y121F/T289A *cyp51A* mutations. Susceptibility testing was performed according to CLSI M38-A2. The MICs of 363 *A. fumigatus* isolates were used in this study. Based on the CLSI epidemiological cut-off values (ECVs), 141 isolates were phenotypically non-WT and 222 isolates had a phenotypically WT susceptibility. All isolates with the TR_34_/L98H mutation had an itraconazole MIC > 1 mg/L which is above the CLSI ECV. Eighty-six of 89 (97%) isolates with the TR_34_/L98H mutation had voriconazole and posaconazole MICs above the CLSI ECV, i.e., MICs of 1 and 0.25 mg/L, respectively. The isolates with a TR_46_/Y121F/T289A mutation showed a different phenotype. All 37 isolates with a TR_46_/Y121F/T289A mutation had a voriconazole MIC above the CLSI ECV, while 28/37 (76%) isolates had an itraconazole MIC > 1 mg/L. Interestingly, only 13 of 37 (35%) isolates had a posaconazole MIC > 0.25 mg/L.

## 1. Introduction

The triazoles, itraconazole, posaconazole, and voriconazole, are antifungal agents with potent activity against *Aspergillus fumigatus*. Voriconazole is predominately used for the treatment of invasive aspergillosis, while posaconazole is used for prophylaxis against invasive fungal infections in patients with hematological diseases, and itraconazole is the first choice for patients with chronic pulmonary aspergillosis. The treatment of *Aspergillus* infections is complicated due to the increase in antifungal drug resistance in *A. fumigatus* in some geographical areas [1,2,3]. Azole resistance in *A. fumigatus* is most frequently conferred by mutations in the *cyp51A* gene encoding 14α-sterol demethylases. TR_34_/L98H and TR_46_/Y121F/T289A are the two most common mutations associated with environmental resistance selection. Importantly, isolates with these environmental associated mutations can be recovered from azole-naïve patients as well as azole-treated patients [4,5,6,7]. Other common mutations such as those at residue M220 or G54 in the *cyp51A* gene were associated with long-term use of azoles in a clinical setting, especially in patients with chronic cavitary aspergillosis [8]. A recent retrospective cohort study showed a 21% increased mortality in patients with azole-resistant invasive aspergillosis compared to azole-susceptible infection [9]. Due to the increase in resistance and the fact that resistance can also be found in azole-naïve patients, susceptibility testing becomes increasingly clinically relevant for guidance of appropriate antifungal therapy [10,11]. Two reference guidelines are available for the susceptibility testing of *A. fumigatus*—broth microdilution based on the Clinical and Laboratory Standards Institute (CLSI) and the European Committee on Antimicrobial Susceptibility Testing (EUCAST) methods [12,13]. The minimal inhibitory concentration (MIC) distributions of an extensive number of wild-type (WT) and azole-resistant isolates were reported with the EUCAST method [14]. However, for the CLSI method, MIC distributions of a large collection of azole-resistant isolates are not available. 

We studied the MIC distributions of clinical *A. fumigatus* isolates to characterize the CLSI susceptibility profiles of isolates with the WT *cyp51A* genotype, and isolates with the TR_34_/L98H and TR_46_/Y121F/T289A *cyp51A* mutations.

## 2. Materials and Methods

Susceptibility data were collected for all clinical isolates cultured in the Canisius-Wilhelmina Hospital (CWZ), Nijmegen, the Netherlands, for which susceptibility testing was performed. Furthermore, all *A. fumigatus* isolates sent from other laboratories to our reference laboratory for susceptibility testing were included in this dataset. The isolates were cultured between 2001 and 2017. Isolates were identified as *A. fumigatus* based on morphological characteristics and the ability to grow at >48 °C.

Susceptibility testing was performed according to CLSI M38-A2. In brief, 96-well plates (with two-fold dilutions of antifungals) were prepared with RPMI-1680 medium with 0.2% glucose. Inoculum suspensions were prepared in sterile 0.9% NaCl with 0.1% Tween 20. Spores were harvested from a mature culture. The suspension was adjusted to 80–82 transmission at 530 nm (Spectrofotometer Genesys 20) to create a 0.4–3.3 × 10^6^ colony-forming units (CFU)/mL of spore suspension [15]. The spore suspensions were added to the microtiter plates to create a final concentration of 0.4 × 10^4^–5 × 10^4^ CFU/mL in each well. Plates were incubated for 48 h at 35 °C. The lowest concentration without visible growth was used as the MIC. The concentration range used was 0.031–16 mg/L for voriconazole, itraconazole, and posaconazole. Susceptibility testing was repeated when non-WT azole MICs were recorded. For quality control of susceptibility testing, *Aspergillus flavus* ATCC 204304, *Candida parapsilosis* ATCC 22019, and *C. krusei* ATCC 6258 were used. The MICs of the reference strains were within the expected range.

The CLSI epidemiological cut-off values (ECVs) used were 1 mg/L for itraconazole, 1 mg/L for voriconazole, and 0.25 mg/L for posaconazole [16,17]. All isolates with an MIC of voriconazole, posaconazole, and/or itraconazole above these ECVs were retested and screened for tandem repeats and point mutations, Gly54, Gly138, and Met220, in the *cyp51A* gene using real-time PCR [18]. When no mutation was identified, the full *cyp51A* was sequenced as previously described [19].

## 3. Results

In total, the susceptibility of 420 isolates was available. Based on the CLSI ECVs, 265 isolates were azole WT and 155 were characterized as non-WT [16,17]. Only one isolate per patient per six months was included unless isolates had different susceptibility results or resistance mechanisms, leaving a total of 363 isolates used in this study. For 33 isolates, no posaconazole MICs were available. Of the 363 isolates, 141 isolates were phenotypically non-WT and 222 isolates had a phenotypically WT susceptibility. In 127 of 141 (90%) phenotypically non-WT isolates, a *cyp51A* gene mutation could be identified; 88/141 (62%) had the TR_34_/L98H mutations [20], 37/141 (26%) had the TR_46_/Y121F/T289A or TR_46_^3^/Y121F/T289A mutation, and one had the TR_53_ mutation [21,22]. One isolate with confirmed azole resistance had an M220V point mutation. G54 and G138 were not found in this clinical isolate collection. For 14/141 (10%) isolates, no *cyp51A* mutations were detected.

**Figure 1:**
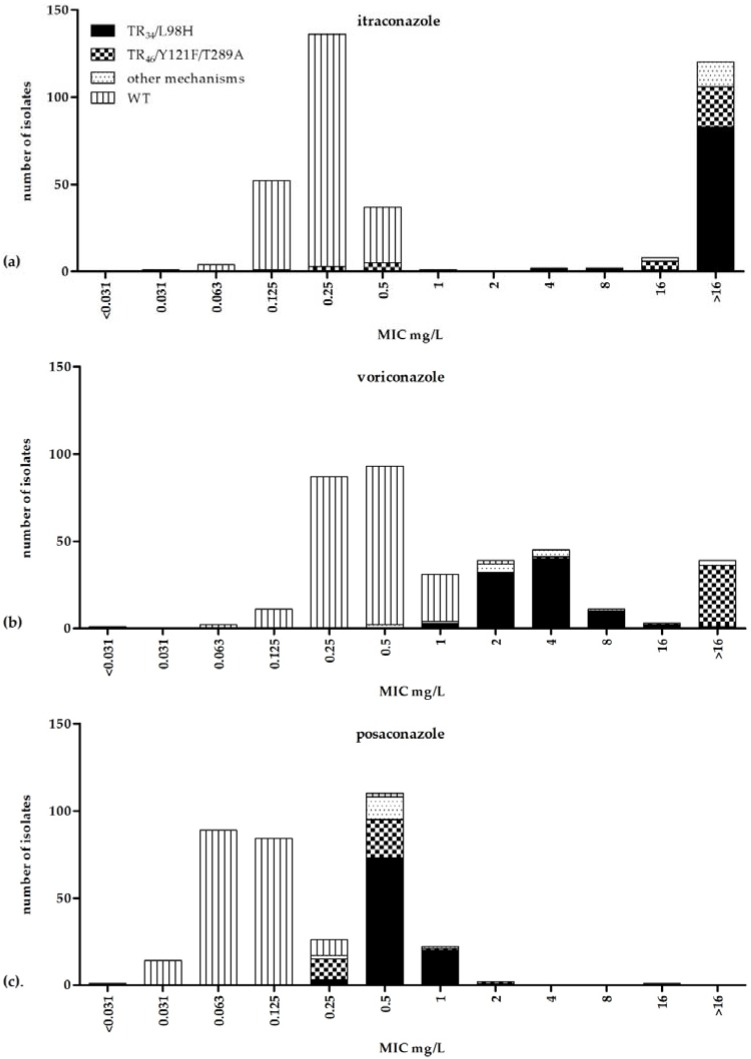
Clinical and Laboratory Standards Institute (CLSI) MIC distribution of 363 clinical isolates of *Aspergillus fumigatus* including 89 isolates with the TR_34_/L98H mutation, 37 with the TR_46_/Y121F/T289A mutation, and 16 isolates with other mechanisms—1 isolate with a *cyp51A* point mutation (M220V), one with a TR53 mutation, and 14 non-*cyp51A* azole resistance mechanisms—and 222 phenotypically wild-type isolates. MIC distributions are displayed for (**a**) itraconazole, (**b**) voriconazole, and (**c**) posaconazole.

The MIC distributions of voriconazole, itraconazole, and posaconazole for isolates with an azole susceptible phenotype are displayed in Figure 1. The MIC_50_ and MIC_90_ values for these isolates are displayed in Table 1. All isolates with the TR_34_/L98H mutation had an itraconazole MIC > 1 mg/L, which is above the CLSI ECV, while 85 of 88 (97%) isolates had a voriconazole above the CLSI ECV of MIC 1 mg/L, and 85 of 88 (97%) isolates had a posaconazole MIC above the CLSI ECV of 0.25 mg/L.

The isolates with a TR_46_/Y121F/T289A mutation showed a different phenotype. All isolates had a voriconazole MIC above the CLSI ECV, while 28/37 (76%) isolates had an MIC > 1 mg/L for itraconazole. Furthermore, 13 of 37 (35%) isolates had a posaconazole MIC > 0.25 mg/L (Figure 1). All isolates with *cyp51A* resistance mutations had an MIC outside the WT range for voriconazole, itraconazole, or posaconazole.

**Figure 2:**
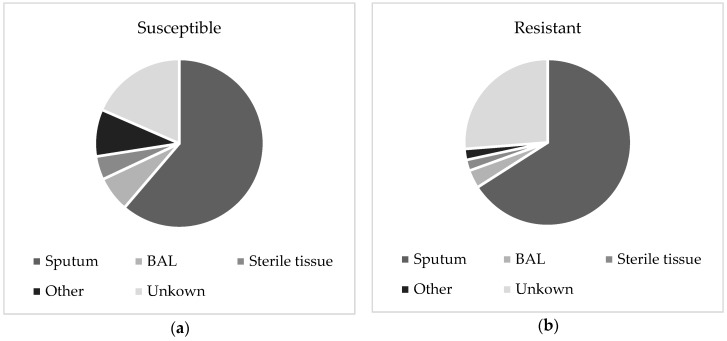
Pie chart showing the distribution of positive *Aspergillus fumigatus* cultures for sputum, bronchoalveolar lavage (BAL), sterile tissue, and other samples. Distributions are shown for susceptible isolates (**a**), and resistant isolates (**b**).

For 285 isolates, the sample origin was available, and the majority were isolated from sputum and bronchial aspirates. Ninety-three of the resistant isolates were cultured from sputum and bronchial aspirate, five were from bronchoalveolar lavage (BAL) samples, three were from sterile tissue biopsies, and for 37 resistant isolates, the origin is unknown. For susceptible isolates, a higher proportion was cultured from BAL or sterile tissues. The distributions are displayed in Figure 2.

## 4. Discussion

As TR_34_/L98H and TR_46_/Y121F/T289A mutations are increasingly found worldwide, it is important to characterize the susceptibility profile of isolates with these mutations. Such a profile was published for the EUCAST method of susceptibility testing [14]. However, data are scarce for the CLSI method. In this study, we characterized the MIC distributions of WT isolates and isolates with the TR_34_/L98H and TR_46_/Y121F/T289A mutations. As expected, the CLSI susceptibility profiles in our study were similar to the EUCAST susceptibility profiles [14]. For both EUCAST and CLSI, most isolates with a TR_34_/L98H mutation had a high (>8 mg/L) MIC of itraconazole, while most of the MICs of voriconazole were between 2 and 16 mg/L [14]. The MICs of posaconazole were between 0.25 and 2 mg/L for both methods. The isolates with the TR_46_/Y121F/T289A mutation showed high MICs of voriconazole (>8 mg/L) for both methods with variable susceptibility for itraconazole, which is in line with a recent compilation of the literature [23]. The MICs of posaconazole were between 0.25 and 4 mg/L for both methods. Compared to WT isolates, the posaconazole MIC seems the least affected by TR_46_/Y121F/T289A and TR_34_/L98H mutations, and the distribution of non-WT isolates overlaps with WT isolates. In vivo models indicate that isolates with an increased posaconazole MIC may be treatable if the posaconazole exposure is increased [24,25]. As the posaconazole MIC remains relatively low and overlap with the WT population exists, azole-resistant aspergillosis might, in some cases, be treated with high posaconazole dosages. Virtually no overlap of MIC distributions was observed for itraconazole and voriconazole; however, some TR_34_/L98H isolates have low voriconazole MICs which are possibly still treatable with voriconazole. In this collection, 35 of 89 (40%) had an MIC <4 mg/L.

The resistance profiles of susceptible and resistant isolates give us information on which azoles to use for resistance screening. When only itraconazole is used for resistance screening, some TR_46_/Y121F/T289A mutations will be missed as 9/37 (24%) had low MICs of itraconazole. In contrast, when only voriconazole is used, a significant proportion of TR_34_/L98H resistance may be missed. As the MIC distributions of WT and non-WT isolates overlap for posaconazole, this agent seems not a good drug for resistance screening. However, all isolates had high levels of resistance for either itraconazole or voriconazole. Therefore, it is advised to use a method applying a combination of itraconazole and voriconazole, with or without the use of posaconazole (using a concentration above the WT MIC).

As the isolates were only screened for *cyp51A*, mutations it is possible that some of the isolates have additional mechanisms of azole drug tolerance, such as mutations in *hapE* [26,27], decreased absorption of azoles [28], or increased expression of efflux pumps [29,30,31]. Possibly, such additional resistance mechanisms explain the broad range of itraconazole MICs found for isolates with the TR_46_/Y121F/T289A mutations, as well as the broad range of voriconazole MICs found for isolates with TR_34_/L98H. Recently, a new resistance mutation was described with a triple TR_46_ promotor repeat [27]. Additional mutations in combination with TR_34_/L98H were reported to be associated with elevated MIC values of imidazole fungicides used in the environment [32]. These findings suggest that the resistance mechanisms are continuously evolving under continued environmental azole pressure. As we used real-time PCR [18] to detect the tandem repeats, and the G54, M220, and G138 mutations, we only sequenced the full *cyp51A* gene when the four mutations were not found, and any additional mutations in combination with TR_34_/L98H, TR_46_/Y121F/T289A, G54, M220, or G138 were not detected. 

For 285 of 363 isolates (79%), the sample origin was known. A higher proportion of susceptible *A. fumigatus* isolates were cultured from sterile tissue and BAL samples compared to resistant isolates. In an unselected collection of *A. fumigatus* isolates, this might reflect a higher virulence for susceptible isolates compared to resistant isolates. However, many isolates were referred to our reference laboratory for susceptibility testing by other laboratories. It is likely that isolates cultured from clinically relevant sites, i.e., sterile tissue or BAL samples, are more likely to be referred for susceptibility testing despite negative azole screening results. On the contrary, isolates that are considered less clinically relevant are only referred when azole screening indicates resistance.

Susceptibility testing using the CLSI or EUCAST reference methods are not available in many laboratories. Therefore, non-specialized laboratories in the Netherlands send *Aspergillus* isolates to the Center of Expertise in Mycology Radboudumc/CWZ for susceptibility testing when indicated. Furthermore, for the surveillance of antifungal resistance, many laboratories in the Netherlands use the VIPcheck™ for screening of azole resistance [33,34]. When the screening plate indicates a possible azole-resistant isolate, the isolates are sent to a mycology reference laboratory for in vitro susceptibility testing. Therefore, the proportion of resistant isolates in this study does not reflect the current resistance rate in the Netherlands. However, the large number of resistant isolates in this study can be used to characterize the susceptibility profile and can be used to identify the most prevalent resistance mechanisms.

In this collection of isolates, the most common azole resistance mechanism was due to the TR_34_/L98H mutation, which was also seen in a previous study with isolates from the Netherlands [14]. The TR_34_/L98H mechanism was the first of the environmental mutations recovered from patients [1]. Since 2009, the TR_46_/Y121F/T289A mutation was also found in clinical samples from the Netherlands, but earlier isolates were found in the USA in 2008 [1,35]. This mechanism of resistance was the second most recovered mechanism. Both resistance mechanisms were correlated with clinical failure in retrospective case studies [1], and accounted for 87% of azole-resistant *A. fumigatus* isolates in a recent cohort study, which showed a 21% excess day-42 mortality in resistance invasive aspergillosis compared with voriconazole-susceptible infection [9].

Recently, resistance detection PCRs became available. AsperGenius, a method for the direct molecular detection of resistance in *A. fumigatus* from clinical materials is commercially available. The AsperGenius is able to identify TR_34_/L98H and TR_46_/Y121F/T289A mutations, and is validated for BAL samples. The test was able to distinguish resistant from susceptible *Aspergillus* from BAL samples, even when cultures were negative. Importantly, the detection of azole resistance with the AsperGenius correlated with azole treatment failure [36]. Furthermore, the AsperGenius was evaluated on plasma and serum samples; however, the sensitivity of resistance detection in these sample types appears to be limited [37]. Another commercial resistance PCR, the MycoGENIE, has the ability to detect TR_34_/L98H mutations. The assay was evaluated on samples from patients with fungal rhinosinusitis, as well as BAL and serum samples [38,39]. Using the susceptibility data of this study, it is possible to predict the susceptibility phenotype of itraconazole for TR_34_/L98H and the phenotype of voriconazole for TR_46_/Y121F/T289A isolates. As voriconazole itraconazole, and posaconazole MICs are variable for TR_34_/L98H and TR_46_/Y121F/T289A isolates using CLSI methods, it is advisable to move away from the azole class if the resistance PCR identifies a mutation, but cultures remain negative. However, the AsperGenius detects the TR_34_/L98H and TR_46_/Y121F/T289A mutations, while the MycoGENIE detects only the TR_34_/L98H mutations, and thus, other resistance mutations will be missed. As resistance mutations in patients with long-term azole use are more diverse and may contain both *cyp51A* and non-*cyp51A* mechanisms, the results of molecular methods should be carefully interpreted [40]. Hence, susceptibility testing remains paramount and molecular methods should be used to supplement phenotypic susceptibility testing.

In conclusion, this study provides data on the susceptibility of *A. fumigatus* isolates using the CLSI method and characterizes the phenotypical antifungal profile of isolates harboring TR_34_/L98H and TR_46_/Y121F/T289A resistance mechanisms. In uncontrolled studies, an increased MIC of azoles, as well as the detection of resistance mutations, is correlated with clinical failure, suggesting that susceptibility testing using CLSI is an important tool to guide antifungal therapy.

## Figures and Tables

**Table 1:** MIC_50_ and MIC_90_ values of itraconazole, voriconazole, and posaconazole for clinical isolates of *Aspergillus fumigatus*.

*Cyp51A* Genotype	Number of Isolates	Itraconazole (mg/L)		Voriconazole (mg/L)		Posaconazole (mg/L)	
		MIC_50_^1^	MIC_90_^1^	MIC_50_^1^	MIC_90_^1^	MIC_50_^1^	MIC_90_^1^
Wild-type isolates ^2^	222	0.25	0.5	0.5	1	0.125	0.125
TR_34_/L98H	89	>16	>16	4	8	0.5	1
TR_46_/Y121F/T289A	37	16	>16	>16	>16	0.5	0.5
other mechanisms	16	>16	>16	2	8	0.5	0.5

^1^ MIC50 and MIC90 values were defined as the lowest concentration of the antifungal at which 50 and 90% of the isolates were inhibited, respectively. ^2^ phenotypically wild-type isolates.

## References

[1] Verweij P.E., Chowdhary A., Melchers W.J., Meis J.F. (2016). Azole resistance in *Aspergillus fumigatus:* Can we retain the clinical use of mold-active antifungal azoles?. Clin. Infect. Dis..

[2] Chowdhary A., Sharma C., Meis J.F. (2017). Azole-resistant aspergillosis: Epidemiology, molecular mechanisms, and treatment. J. Infect. Dis..

[3] Resendiz Sharpe A., Lagrou K., Meis J.F., Chowdhary A., Lockhart S.R., Verweij P.E. (2018). Triazole resistance surveillance in *Aspergillus fumigatus*. Med. Mycol..

[4] Pelaez T., Monteiro M.C., Garcia-Rubio R., Bouza E., Gomez-Lopez A., Mellado E. (2015). First detection of *Aspergillus fumigatus* azole-resistant strain due to *cyp51A* TR_46_/Y121F/T289A in an azole-naive patient in Spain. New Microbes New Infect..

[5] van der Linden J.W., Camps S.M., Kampinga G.A., Arends J.P., Debets-Ossenkopp Y.J., Haas P.J., Rijnders B.J., Kuijper E.J., van Tiel F.H., Varga J. (2013). Aspergillosis due to voriconazole highly resistant *Aspergillus fumigatus* and recovery of genetically related resistant isolates from domiciles. Clin. Infect. Dis..

[6] van der Linden J.W., Snelders E., Kampinga G.A., Rijnders B.J., Mattsson E., Debets-Ossenkopp Y.J., Kuijper E.J., Van Tiel F.H., Melchers W.J., Verweij P.E. (2011). Clinical implications of azole resistance in *Aspergillus fumigatus*, the Netherlands, 2007–2009. Emerg. Infect. Dis..

[7] Meis J.F., Chowdhary A., Rhodes J.L., Fisher M.C., Verweij P.E. (2016). Clinical implications of globally emerging azole resistance in *Aspergillus fumigatus*. Phil. Trans. R. Soc. Lond. B Biol. Sci..

[8] Howard S.J., Cerar D., Anderson M.J., Albarrag A., Fisher M.C., Pasqualotto A.C., Laverdiere M., Arendrup M.C., Perlin D.S., Denning D.W. (2009). Frequency and evolution of azole resistance in *Aspergillus fumigatus* associated with treatment failure. Emerg. Infect. Dis..

[9] Lestrade P.P., Bentvelsen R.G., Schauwvlieghe A.F.A.D., Schalekamp S., van der Velden W.J., Kuiper E.J., van Paassen J., van der Hoven B., van der Lee H.A., Melchers W.J. (2018). Voriconazole resistance and mortality in invasive aspergillosis: A multicenter retrospective cohort study. Clin. Infect. Dis..

[10] Ullmann A.J., Aguado J.M., Arikan-Akdagli S., Denning D.W., Groll A.H., Lagrou K., Lass-Flörl C., Lewis R.E., Munoz P., Verweij P.E. (2018). Diagnosis and management of *Aspergillus* diseases: Executive summary of the 2017 ESCMID-ECMM-ERS guideline. Clin. Microbiol. Infect..

[11] Verweij P.E., Ananda-Rajah M., Andes D., Arendrup M.C., Bruggemann R.J., Chowdhary A., Cornely O.A., Denning D.W., Groll A.H., Izumikawa K. (2015). International expert opinion on the management of infection caused by azole-resistant *Aspergillus fumigatus*. Drug Resist. Updat..

[12] Clinical and Laboratory Standards Institute (CLSI) (2017). Reference Method for Broth Dilution Antifungal Susceptibility Testing of Filamentous Fungi.

[13] European Committee for Antimicrobial Susceptibility Testing (EUCAST) (2015). Method for the Determination of Broth Dilution Minimum Inhibitory Concentrations of Antifungal Agents for Conidia Forming Moulds.

[14] van Ingen J., van der Lee H.A., Rijs T.A., Zoll J., Leenstra T., Melchers W.J., Verweij P.E. (2015). Azole, polyene and echinocandin mic distributions for wild-type, TR34/L98H and TR46/Y121F/T289A *Aspergillus fumigatus* isolates in the Netherlands. J. Antimicrob. Chemother..

[15] Espinel-Ingroff A., Dawson K., Pfaller M., Anaissie E., Breslin B., Dixon D., Fothergill A., Paetznick V., Peter J., Rinaldi M. (1995). Comparative and collaborative evaluation of standardization of antifungal susceptibility testing for filamentous fungi. Antimicrob. Agents Chemother..

[16] Meletiadis J., Mavridou E., Melchers W.J., Mouton J.W., Verweij P.E. (2012). Epidemiological cutoff values for azoles and *Aspergillus fumigatus* based on a novel mathematical approach incorporating *Cyp51A* sequence analysis. Antimicrob. Agents Chemother..

[17] Espinel-Ingroff A., Diekema D.J., Fothergill A., Johnson E., Pelaez T., Pfaller M.A., Rinaldi M.G., Canton E., Turnidge J. (2010). Wild-type MIC distributions and epidemiological cutoff values for the triazoles and six *Aspergillus* spp. For the CLSI broth microdilution method (M38-A2 document). J. Clin. Microbiol..

[18] Klaassen C.H., de Valk H.A., Curfs-Breuker I.M., Meis J.F. (2010). Novel mixed-format real-time PCR assay to detect mutations conferring resistance to triazoles in *Aspergillus fumigatus* and prevalence of multi-triazole resistance among clinical isolates in the Netherlands. J. Antimicrob. Chemother..

[19] Sharma C., Hagen F., Moroti R., Meis J.F., Chowdhary A. (2015). Triazole-resistant *Aspergillus fumigatus* harbouring G54 mutation: Is it de novo or environmentally acquired?. J. Glob. Antimicrob. Resist..

[20] Mellado E., Garcia-Effron G., Alcazar-Fuoli L., Melchers W.J., Verweij P.E., Cuenca-Estrella M., Rodriguez-Tudela J.L. (2007). A new *Aspergillus fumigatus* resistance mechanism conferring in vitro cross-resistance to azole antifungals involves a combination of *cyp51A* alterations. Antimicrob. Agents Chemother..

[21] Zhang J., Snelders E., Zwaan B.J., Schoustra S.E., Meis J.F., van Dijk K., Hagen F., van der Beek M.T., Kampinga G.A., Zoll J. (2017). A novel environmental azole resistance mutation in *Aspergillus fumigatus* and a possible role of sexual reproduction in its emergence. mBio.

[22] Hodiamont C.J., Dolman K.M., Ten Berge I.J., Melchers W.J., Verweij P.E., Pajkrt D. (2009). Multiple-azole-resistant *Aspergillus fumigatus* osteomyelitis in a patient with chronic granulomatous disease successfully treated with long-term oral posaconazole and surgery. Med. Mycol..

[23] Dudakova A., Spiess B., Tangwattanachuleeporn M., Sasse C., Buchheidt D., Weig M., Groß U., Bader O. (2017). Molecular Tools for the Detection and Deduction of Azole Antifungal Drug Resistance Phenotypes in *Aspergillus* Species. Clin. Microbiol. Rev..

[24] Lepak A.J., Marchillo K., Vanhecker J., Andes D.R. (2013). Posaconazole pharmacodynamic target determination against wild-type and *cyp*51 mutant isolates of *Aspergillus fumigatus* in an in vivo model of invasive pulmonary aspergillosis. Antimicrob. Agents Chemother..

[25] Mavridou E., Bruggemann R.J., Melchers W.J., Mouton J.W., Verweij P.E. (2010). Efficacy of posaconazole against three clinical *Aspergillus fumigatus* isolates with mutations in the *cyp51A* gene. Antimicrob. Agents Chemother..

[26] Camps S.M., Dutilh B.E., Arendrup M.C., Rijs A.J., Snelders E., Huynen M.A., Verweij P.E., Melchers W.J. (2012). Discovery of a hape mutation that causes azole resistance in *Aspergillus fumigatus* through whole genome sequencing and sexual crossing. PLoS ONE.

[27] Gsaller F., Hortschansky P., Furukawa T., Carr P.D., Rash B., Capilla J., Muller C., Bracher F., Bowyer P., Haas H. (2016). Sterol biosynthesis and azole tolerance is governed by the opposing actions of SrbA and the CCAAT binding complex. PLoS Pathog..

[28] Wei X., Chen P., Gao R., Li Y., Zhang A., Liu F., Lu L. (2017). Screening and characterization of a non-*cyp51A* mutation in an *Aspergillus fumigatus cox*10 strain conferring azole resistance. Antimicrob. Agents Chemother..

[29] Fraczek M.G., Bromley M., Buied A., Moore C.B., Rajendran R., Rautemaa R., Ramage G., Denning D.W., Bowyer P. (2013). The *cdr*1b efflux transporter is associated with non-*cyp51A*-mediated itraconazole resistance in *Aspergillus fumigatus*. J. Antimicrob. Chemother..

[30] Paul S., Diekema D., Moye-Rowley W.S. (2013). Contributions of *Aspergillus fumigatus* atp-binding cassette transporter proteins to drug resistance and virulence. Eukaryot. Cell.

[31] Meneau I., Coste A.T., Sanglard D. (2016). Identification of *Aspergillus fumigatus* multidrug transporter genes and their potential involvement in antifungal resistance. Med. Mycol..

[32] Chen Y., Li Z., Han X., Tian S., Zhao J., Chen F., Su X., Zhao J., Zou Z., Gong Y. (2018). Elevated MIC values of imidazole drugs against *Aspergillus fumigatus* isolates with TR34/L98H/A297T/F495I mutation. Antimicrob. Agents Chemother..

[33] Buil J.B., van der Lee H.A.L., Rijs A., Zoll J., Hovestadt J., Melchers W.J.G., Verweij P.E. (2017). Single-center evaluation of an agar-based screening for azole resistance in *Aspergillus fumigatus* by using vipcheck. Antimicrob. Agents Chemother..

[34] Arendrup M.C., Verweij P.E., Mouton J.W., Lagrou K., Meletiadis J. (2017). Multicentre validation of 4-well azole agar plates as a screening method for detection of clinically relevant azole-resistant *Aspergillus fumigatus*. J. Antimicrob. Chemother..

[35] Wiederhold N.P., Gil V.G., Gutierrez F., Lindner J.R., Albataineh M.T., McCarthy D.I., Sanders C., Fan H., Fothergill A.W., Sutton D.A. (2016). First detection of TR34 L98H and TR46 Y121F T289A *cyp*51 mutations in *Aspergillus fumigatus* isolates in the United States. J. Clin. Microbiol..

[36] Chong G.M., van der Beek M.T., von dem Borne P.A., Boelens J., Steel E., Kampinga G.A., Span L.F., Lagrou K., Maertens J.A., Dingemans G.J. (2016). PCR-based detection of *Aspergillus fumigatus cyp51A* mutations on bronchoalveolar lavage: A multicentre validation of the AsperGenius assay^(r)^ in 201 patients with haematological disease suspected for invasive aspergillosis. J. Antimicrob. Chemother..

[37] Buil J.B., Zoll J., Verweij P.E., Melchers W.J.G. (2018). Molecular detection of azole-resistant *Aspergillus fumigatus* in clinical samples. Front. Microbiol..

[38] Morio F., Dannaoui E., Chouaki T., Cateau E., Malard O., Bonfils P., Page C., Dufour X., Cottrel C., Erwan T. (2018). PCR-based detection of *Aspergillus fumigatus* and absence of azole resistance due to TR_34_/L98H in a French multicenter cohort of 137 patients with fungal rhinosinusitis. Mycoses.

[39] Dannaoui E., Gabriel F., Gaboyard M., Lagardere G., Audebert L., Quesne G., Godichaud S., Verweij P.E., Accoceberry I., Bougnoux M.E. (2017). Molecular diagnosis of invasive aspergillosis and detection of azole resistance by a newly commercialized PCR kit. J. Clin. Microbiol..

[40] Ballard E., Melchers W.J.G., Zoll J., Brown A.J.P., Verweij P.E., Warris A. (2018). In-host microevolution of *Aspergillus fumigatus*: A phenotypic and genotypic analysis. Fungal Genet. Biol..

